# Comparative Histopathological Characteristics of Duodenal Involvement in Different Types of Amyloidosis

**DOI:** 10.3390/biomedicines13092196

**Published:** 2025-09-08

**Authors:** Anna Tebenkova, Zarina Gioeva, Nikolay Shakhpazyan, Valentina Pechnikova, Konstantin Midiber, Vladislav Kalmykov, Liudmila Mikhaleva

**Affiliations:** Avtsyn Research Institute of Human Morphology of Federal State Budgetary Scientific Institution “Petrovsky National Research Centre of Surgery”, 117418 Moscow, Russia; tebenkova.ann@yandex.ru (A.T.); nshakhpazyan@gmail.com (N.S.); valiagtx@yandex.ru (V.P.); midiberkonst@gmail.com (K.M.); xxor2011@gmail.com (V.K.); mikhalevalm@yandex.ru (L.M.)

**Keywords:** systemic amyloidosis, duodenal amyloidosis, gastrointestinal tract, immunohistochemistry, histopathology, biopsy, autopsy

## Abstract

**Background/Objectives**: The duodenum is commonly involved in systemic amyloidosis. This retrospective observational study describes histoanatomical distributions of different types of duodenal amyloidosis to improve the diagnostic value of duodenal biopsies. **Methods**: We examined 21 biopsy and 16 autopsy specimens from duodenal amyloidosis patients. Immunohistochemical typing was performed using a broad panel of antibodies against different amyloid types. **Results**: AL lambda amyloidosis was determined in 5 (13%) biopsies and 7 (18%) autopsies, exhibiting interstitial and intravascular amyloid deposition patterns in 11 (92%) cases; AL kappa amyloidosis—in 7 (18%) biopsies and 1 (3%) autopsy, presenting with a combined interstitial and intravascular deposition pattern in 6 (75%) cases; transthyretin amyloidosis—in 2 (5%) biopsies and 2 (5%) autopsies, showing focal interstitial and intravascular deposits; and AA amyloidosis—in 7 (19%) biopsies and 6 (16%) autopsies, demonstrating a combined pattern of amyloid deposition. Regardless of the specific amyloid type, in 33 (89%) of 37 cases, amyloid deposits were determined in the muscularis mucosae and submucosa of the small intestine, while in the lamina propria, amyloid depositions were found only in 29 (78%) cases. **Conclusions**: When diagnosing duodenal amyloidosis, superficial biopsies can lead to false negative results. This is particularly true for ATTR amyloidosis, where mucosal involvement is rare. The most extensive amyloid deposits were observed in AL kappa amyloidosis. Gastrointestinal bleeding was a more frequent complication of AA amyloidosis stemming from the extensive amyloid deposits within the lamina propria which cause vascular fragility and friability.

## 1. Introduction

Amyloidosis is a disease characterized by the abnormal deposition of β-sheet fibrillar proteins, known as amyloid fibrils, in various tissues and organs. The gastrointestinal (GI) tract is commonly affected in patients with systemic or localized amyloidosis. In most cases it can present without noticeable symptoms for a considerable period and often becomes an unexpected finding [1].

According to the most recently updated amyloid nomenclature, the total number of human amyloid fibril proteins is 42; 13 of them may affect the GI tract, 10 are associated with systemic deposition, 3 with localized, and 2 with either systemic or localized deposition (Table 1) [2].

AL amyloidosis is the most common type of amyloidosis. Its GI endoscopic findings include polypoid protrusions, thickened Kerckring folds, submucosal hematomas, and ulcers. Patients with AL amyloidosis may present with GI issues such as constipation, pseudo-obstruction, and bleeding [3].

AA amyloidosis is the second most common type of amyloidosis after AL amyloidosis. AA amyloidosis is more frequently associated with grain-like amyloid deposits in the GI mucosa, leading to its fragility, friability, and erosions. This can result in typical symptoms like diarrhea, malabsorption, weight loss, and GI bleeding [4,5].

In transthyretin amyloidosis (ATTR), GI involvement is considered rare and characterized by a lack of endoscopic findings. Key clinical manifestations of ATTR amyloidosis are transthyretin amyloid cardiomyopathy and polyneuropathy, while GI symptoms are less prominent [6,7].

GI involvement in amyloidosis can range from 3.2% to 36.5% depending on the study and the type of amyloidosis [8,9,10]. It is challenging to diagnose GI amyloidosis because its clinical presentation is often non-specific [11].

The diagnosis of amyloidosis can be definitively established only through histological examination of biopsy samples [12]. When amyloid deposits are stained with hematoxylin and eosin (H&E), they appear as homogenous eosinophilic structures, and after staining with Congo red, they show a characteristic birefringence in polarized light. However, the types and characteristics of amyloid fibrils (e.g., full length, thickness, fragmentation into shorter units) can influence the intensity of birefringence and make it more challenging to diagnose some types of amyloidosis, particularly ATTR [13].

In pathology samples, the color often varies significantly, and there is usually a mixture of green, red, orange, and yellow. Therefore, the International Society of Amyloidosis (ISA) Nomenclature Committee, at its meeting in 2024, recommended the use of a more correct description, such as “characteristic birefringence”, which is replacing the older term “apple-green birefringence” [2].

While in the past rectal biopsy was the most common method for diagnosing systemic amyloidosis, in recent decades, in view of the more frequent use of GI biopsies, duodenal biopsy has been considered a more reliable and preferred diagnostic approach for verifying the disease [14,15,16].

Scientific publications indicate that certain types of amyloidosis exhibit distinct characteristics depending on patient gender and age, histoanatomical distribution of amyloid deposits, and the prominent deposition pattern in the GI tract [17]. In view of the above data, it is crucial to understand the clinical features, morphological characteristics, and histoanatomical distribution of amyloid deposits across different types of duodenal amyloidosis to improve the diagnostic value of biopsies.

## 2. Materials and Methods

This retrospective observational study includes 37 cases of duodenal amyloidosis (21 biopsies and 16 autopsies), collected from January 2016 through February 2025, at the Central Pathology Laboratory of Avtsyn Research Institute of Human Morphology of Federal State Budgetary Scientific Institution “Petrovsky National Research Centre of Surgery”. The study was conducted in accordance with the Declaration of Helsinki and approved by the Institutional Ethics Committee of Avtsyn Research Institute of Human Morphology of Federal State Budgetary Scientific Institution “Petrovsky National Research Centre of Surgery,” Minutes No 8 of 29 September 2023 and No 6 of 21 June 2024.

The inclusion criteria for selecting cases included in the study comprised confirmation of duodenal amyloidosis by histopathology. In all biopsy cases, the diagnosis was not established before taking biopsy samples. The biopsy was used to make a diagnosis of amyloidosis.

Biopsy and autopsy samples were fixed with 10% neutral buffered formalin solution and paraffin-embedded according to the standard protocols. Histological sections with a thickness of 3–4 µm were prepared, stained with H&E and Congo red, and then examined using both light microscopy and polarized light microscopy.

Immunohistochemical (IHC) amyloid typing was performed with polyclonal antibodies against amyloid P-component and transthyretin (Cloud-Clone Corp., Katy, TX, USA), monoclonal antibodies against AL kappa (clone CH15, Leica Biosystems, Novocastra™, Newcastle Upon Tyne, UK), and AL lambda (clone SHL53, Leica Biosystems, Novocastra™). Immunostaining was done on formalin-fixed and paraffin-embedded sections with Bond Max Leica immunostainer using the Bond Polymer Refine Red Detection Kit or Bond Polymer Refine Detection (HRP-DAB) (Leica Microsystems, Wetzlar, Germany). Antigen retrieval was carried out with ER2-Bond Epitope Retrieval Solution 2.

## 3. Results

Biopsy and autopsy specimens from 21 men (56.8%) and 16 women (43.2%) aged 32–93 years (a mean age of 65 years) were examined.

**Figure 1:**
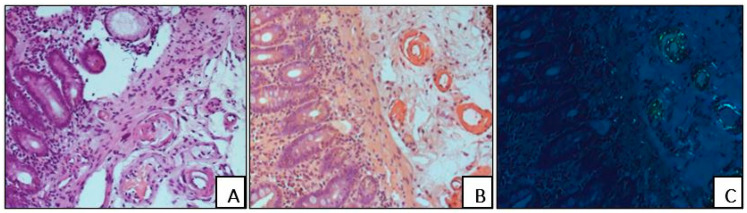
Homogenous eosinophilic amyloid deposition in the wall of the duodenal blood vessels (**A**), H&E staining. Brick-red staining reaction of amyloid deposits in the blood vessels (**B**), Congo red staining. Characteristic birefringence of amyloid masses under polarized light following Congo red staining (**C**). (**A**–**C**) ×200.

In all cases, amyloid deposits were identified with H&E stain as homogenous eosinophilic structures. They demonstrated a brick-red staining reaction and a characteristic birefringence under polarized light following Congo red staining (Figure 1).

**Figure 2:**
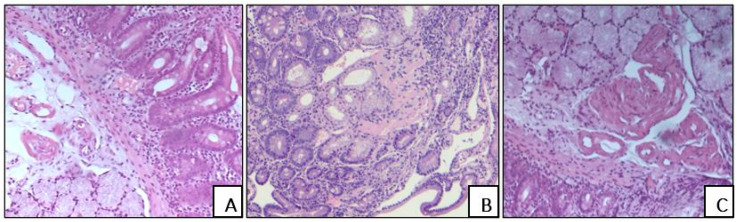
Diverse patterns of amyloid deposition in the small intestine wall, H&E staining, ×200: intravascular (**A**), ×100; interstitial (**B**), ×40; combined intravascular and interstitial (**C**), ×100.

In the duodenal wall, amyloid deposits exhibited diverse patterns: either solely intravascular or combined (interstitial and intravascular) amyloid deposits (Figure 2).

**Figure 3:**
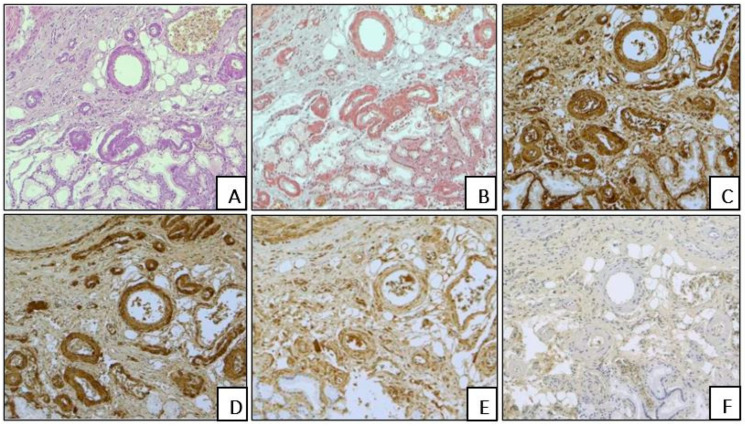
The duodenum of patient B., 75 years old, with AL lambda amyloidosis (an autopsy specimen). Interstitial and intravascular amyloid deposition, H&E (**A**) and Congo red (**B**) staining. A positive IHC reaction with an antibody against amyloid P component (**C**) and against AL lambda amyloid (**D**). A weak background IHC reaction with an anti-AL kappa amyloid antibody (**E**). A negative IHC reaction with an antibody against AA amyloid (**F**). (**A**–**F**) ×100.

Based on IHC amyloid typing, AL lambda amyloid was determined in five (13.5%) biopsies and seven (18.9%) autopsies. Interstitial amyloid deposition was revealed in one (8%) case, while both interstitial and intravascular depositions were found in eleven (92%) cases (Figure 3).

**Figure 4:**
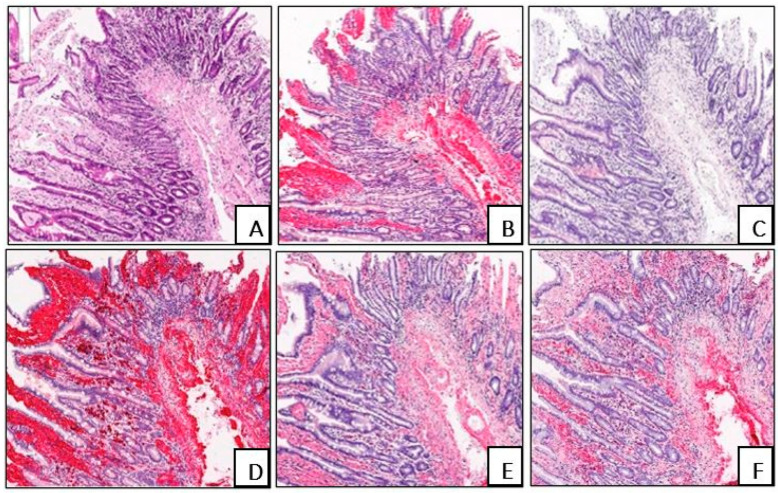
Duodenal biopsy specimen of patient C., 69 years old, with AL kappa amyloidosis. Homogenous acellular interstitial and intravascular amyloid deposits, H&E staining (**A**). A positive IHC reaction with an antibody against amyloid P component (**B**). A negative IHC reaction with an antibody against AA amyloid (**C**). A positive IHC reaction with an antibody against AL kappa amyloid (**D**). A weak background IHC reaction with an anti-AL lambda amyloid antibody (**E**). A negative IHC reaction with an antibody against ATTR (**F**). (**A**–**F**) ×40.

AL kappa amyloid was identified in seven (18.9%) biopsies and one (2.7%) autopsy, presenting with a strong combined interstitial and intravascular deposition pattern in six (75%) cases, an intravascular deposition in six (75%), and only an interstitial deposition in two (25%) cases (Figure 4).

**Figure 5:**
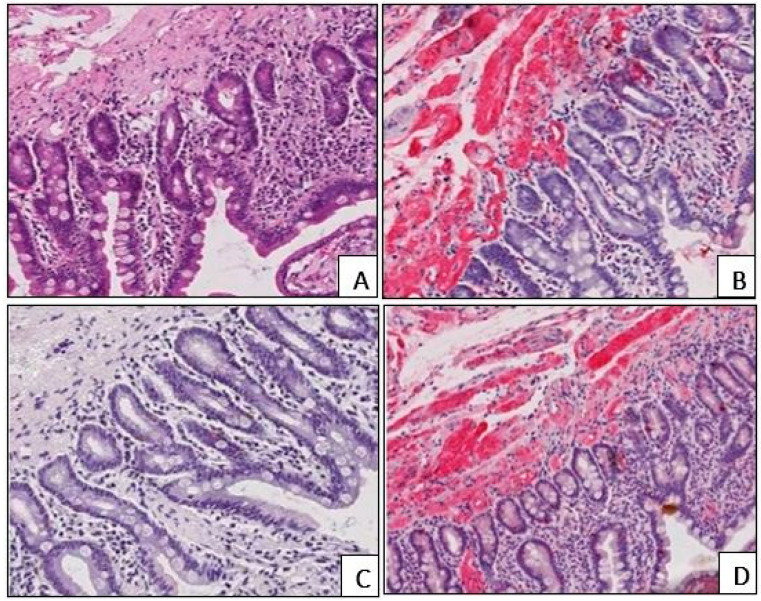
Biopsy specimen of the small intestine of patient A., 72 years old, with transthyretin amyloidosis. Homogenous eosinophilic masses observed under H&E staining (**A**). A positive IHC reaction with an antibody against amyloid P component (**B**) and transthyretin (**D**). A negative IHC reaction against AA amyloid (**C**). (**A**–**D**) ×100.

ATTR was detected in two (5.4%) biopsy and two (5.4%) autopsy samples. In three (75%) of these cases, the amyloid deposition foci were distributed in the interstitial and intravascular tissues, and in one (25%) case, only in the intravascular space (Figure 5).

AA amyloidosis was confirmed in seven (18.9%) biopsy and six (16.2%) autopsy specimens. In all 13 (100%) cases, a combined pattern of amyloid deposition was found.

**Figure 6:**
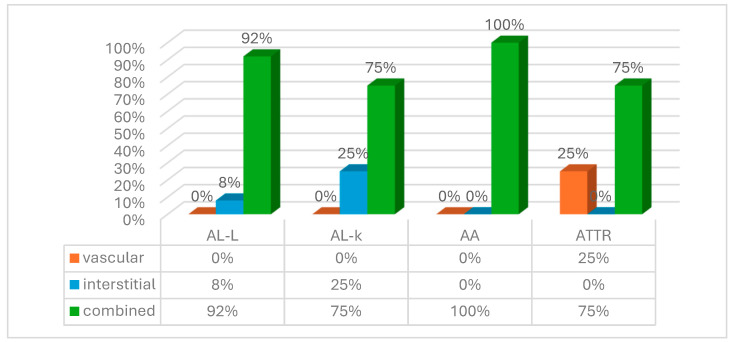
The location of amyloid deposits in the duodenal wall.

Although in most cases a combined pattern of amyloid deposition was observed, solely intravascular amyloid deposits were revealed only in patients with ATTR. No significant differences in the location of amyloid deposits were observed between AL lambda and AL kappa types (Figure 6).

The analysis of the histoanatomical distribution of different amyloid types in the duodenum demonstrated that the lamina propria of the mucous membrane was affected in all five (100%) cases of AL lambda amyloidosis. In the autopsy material, amyloid protein deposits in the lamina propria were detected in five (71%) of seven specimens.

The most intense amyloid depositions were revealed in patients with AL kappa amyloidosis. In 100% of duodenal biopsies and autopsies, amyloid was deposited in the muscularis mucosae, while in the biopsy specimens, the lamina propria of the mucous membrane and the submucosa were affected less frequently—in five (71%) cases.

In patients with AA amyloidosis, amyloid deposits were found in all layers of the small intestine wall, with the same frequency in biopsy and autopsy specimens (seven (100%) and six (100%) cases, respectively).

**Figure 7:**
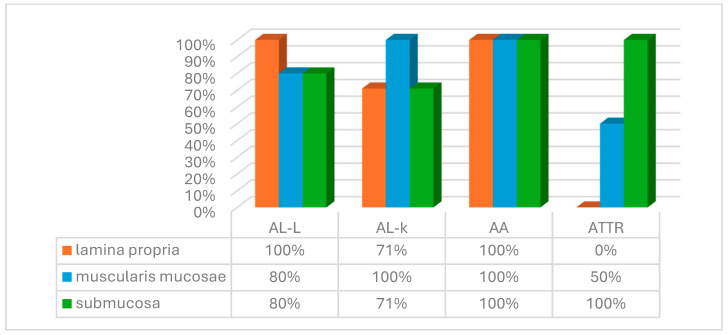
Histoanatomical distribution of amyloid deposits in biopsy samples of the duodenal wall.

**Figure 8:**
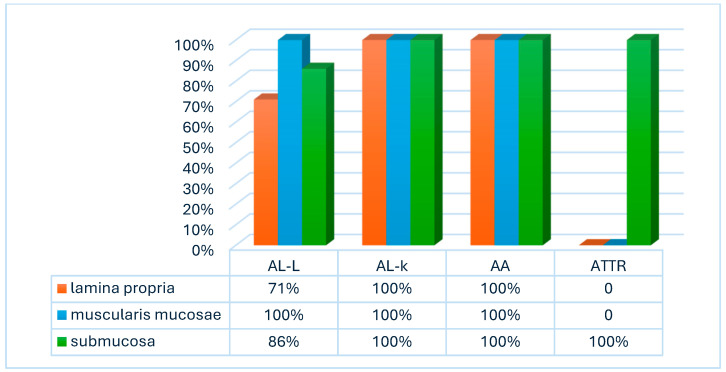
Histoanatomical distribution of amyloid deposits in autopsy samples of the duodenal wall.

In biopsy and autopsy specimens from patients with ATTR, amyloid deposits were not detected in the lamina propria of the mucous membrane, but the submucosa was affected in all cases. The muscularis mucosae was involved in the pathological process in one (25%) biopsy specimen (Figure 7 and Figure 8).

**Figure 9:**
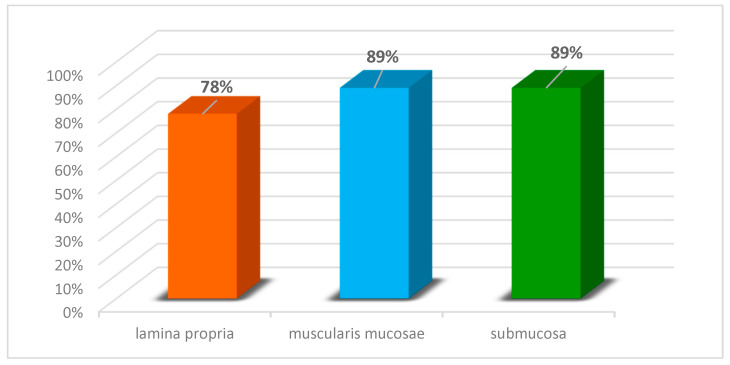
Histoanatomical distribution of amyloid deposits in the duodenum, regardless of the specific amyloid type.

Regardless of the specific amyloid type, in 33 (89%) of 37 studied cases, amyloid deposits were determined in the muscularis mucosae and submucosa of the duodenum, while in the lamina propria of the mucosa, depositions were observed only in 29 (78%) cases (Figure 9).

Correlation analysis assessing the relationship between age and gender of the patients has demonstrated that the diagnosis of duodenal AL amyloidosis was established in 12 (32%) patients during their seventh decade of life. The evaluation of biopsy samples revealed that the most elderly group of patients (mean age, 67 years) had AL lambda amyloidosis, which was followed by AL kappa amyloidosis (mean age, 63 years), AA (mean age, 62 years), and ATTR amyloidosis (mean age, 58 years). The review of autopsy cases indicated that the oldest patients (mean age, 91 years) had ATTR amyloidosis, which was followed by AL lambda amyloidosis (mean age, 68 years), AL kappa (mean age, 68 years), and AA amyloidosis (mean age, 62 years) (Table 2).

**Figure 10:**
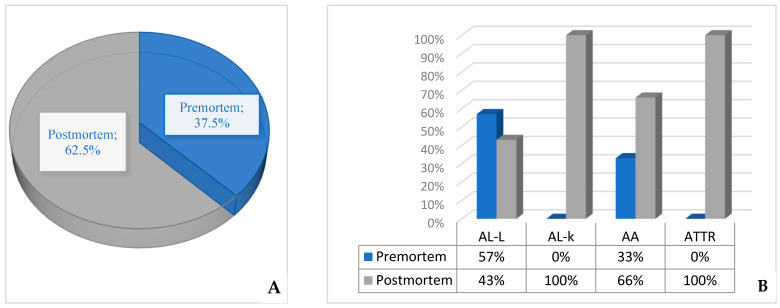
(**A**)—The percentage of systemic amyloidosis cases diagnosed before death (clinical data) compared to those diagnosed after death (autopsy findings). (**B**)—The percentage of cases diagnosed before death (clinical data) compared to those diagnosed after death (autopsy findings), depending on the specific type of amyloidosis.

The analysis of clinical data of autopsy patients indicated that during life, the diagnosis was established only in 6 (37.5%) of 16 patients, including 4 (57%) cases of AL lambda and 2 (33%) of AA amyloidosis. However, AL kappa and ATTR amyloidosis, in all cases, were discovered only at the postmortem examination (Figure 10).

The examination of autopsy specimens revealed that systemic AL amyloidosis was the primary diagnosis in four cases. AL amyloidosis developed as a pre-existing or concurrent disease in patients with multiple myeloma (n = 2), essential hypertension, bilateral nephrosclerosis (n = 1), and COVID-19 (n = 1).

AA amyloidosis was detected in patients with the following diseases: type 2 diabetes mellitus (n = 2), rheumatoid arthritis (n = 1), COVID-19 (n = 1), duodenal ulcer bleeding (n = 1), and cholelithiasis and obstruction of the biliary tract (n = 1).

Patients with determined ATTR amyloidosis had the following underlying diseases: combined atherosclerotic mitral and aortic valve disease (n = 1) and coronavirus disease (COVID-19) (n = 1).

In the studied autopsy cases, GI bleeding was the most frequent clinical manifestation of GI amyloidosis. It was recorded in 5 (31%) of 16 cases (in four (25%) patients with AA amyloidosis and one patient (6%) with AL kappa amyloidosis).

Other clinical signs and symptoms of GI amyloidosis comprised weakness in six (37.5%) cases, weight loss in three (19%), focal ischemia of the small bowel in two (13%), decreased appetite in two (13%), vomiting in one (6%), arterial hypotension in one (6%), and diarrhea in one (6%) patient.

## 4. Discussion

Systemic amyloidosis is often difficult to diagnose, not only because the interpretation of histopathological findings can be complex, but also due to its variable and nonspecific symptoms. Thus, it can be challenging to detect the disease in its early stages [18].

The Amyloidosis Research Consortium has reviewed data on the challenges in amyloidosis diagnosis from the survey completed by 533 participants. For more than a third of respondents, the diagnosis of amyloidosis was not established until ≥1 year after the onset of initial symptoms, and in some cases, the diagnosis was received only after visits to ≥5 physicians [19].

According to the research of Niu Z et al., the overall ante-mortem detection rate of GI amyloidosis in biopsy samples was only 62.8% [16]. In the study carried out by Murat Bektas et al., GI involvement was revealed in 23% of 174 patients with systemic amyloidosis [20]. The research study performed at the UK National Amyloidosis Center has demonstrated that the diagnostic sensitivity of GI biopsy was 75% in patients with amyloidosis and underlying Alzheimer’s disease [21].

Based on data from the Transthyretin Amyloidosis Outcomes Survey (THAOS) registry, 63% of patients with ATTRv experienced GI symptoms at the time of hospitalization. However, in patients with ATTRwt primarily exhibiting cardiac involvement, GI symptoms were reported only in 15% of cases, since cardiac issues are the dominant manifestation in this group of patients [22].

In our research, the evaluation of biopsy specimens demonstrated that most patients had AA amyloidosis (54%) and AL kappa (88%) amyloidosis, while AL amyloidosis (54%) was the most frequently detected type in the performed autopsy studies, suggesting a delayed diagnosis and a lack of treatment provided to patients with AL lambda amyloidosis. Thus, it is suggested that patients with AL amyloidosis often experience delayed diagnosis and may not receive adequate treatment.

Our study found that 71% of amyloidosis cases identified through ante-mortem histological studies were in male patients, while 63% of amyloidosis cases detected at autopsy were in females. The review of autopsy cases indicated that during life, the diagnosis was established only in 37.5% of patients who had amyloidosis. Several studies elucidated that GI symptoms and signs of systemic amyloidosis are more prevalent in older patients [10,23]. Our research has demonstrated that 68% of the studied individuals were around the age of 60 or older.

The analysis of autopsy studies demonstrated that ATTR amyloidosis was the most common diagnosis among the oldest patients (mean age of 91 years). However, the studied patients with the lowest mean age also had ATTR amyloidosis detected through biopsy (the lowest age in this patient group was 58 years). It is suggested that these patients could have ATTRv. However, AA amyloidosis was detected in the biopsy sample taken from the youngest (32-year-old) patient.

Miklos Bely et al. specifically noted that amyloid deposits in the GI tract may develop with equal frequency in both male and female patients and can occur at any stage of rheumatoid arthritis [24].

The assessment of amyloid deposition patterns has shown that in patients with ATTR amyloidosis, the amyloid deposits were not solely found in the interstitial space of the GI tract. This is consistent with the publication of Freudenthaler et al., which states that ATTR amyloidosis with a solely interstitial pattern in the GI tract is rare [17]. Histopathological findings in GI amyloidosis cases, described by Krauß LU et al., also confirmed that the (peri)vascular pattern of amyloid deposition was more common (78.3% of cases) than the interstitial pattern (43.5% of cases) [10].

GI bleeding is one of the most frequent manifestations of GI amyloidosis. In the study by Krauß LU et al., GI bleeding was observed in nine (39.1%) patients with AL amyloidosis, but only two (22.2%) of those with AL amyloidosis required emergency hospitalization due to the bleeding [10]. Hagen CE et al. found that GI bleeding occurred in 21.2% of cases [1].

GI bleeding identified through clinical records occurred in five (31%) patients (four with AA and one with AL kappa amyloidosis) who underwent autopsy examinations within our study. In all cases of AA amyloidosis, histopathological findings included massive amyloid deposits throughout the small bowel wall, leading to vascular friability, focal ischemia, and bleeding.

In general, GI bleeding in systemic AL amyloidosis occurs less frequently than in AA amyloidosis, with estimates suggesting it affects about 15% of patients with AL amyloidosis [22]. In the study by Müller C et al. focusing on AL amyloidosis patients with upper GI bleeding, the most common symptoms leading to hospitalization were melena (38%), hematemesis (35%), and epigastric pain (19%) [25].

For accurate diagnosis of GI involvement in systemic amyloidosis, understanding the distribution of amyloid deposits across different types is crucial, as different types of amyloidosis exhibit varying patterns of amyloid deposition in the GI tract. In routine duodenal biopsies, the tissue sample often only includes the lamina propria of the mucous membrane, which is insufficient to definitively rule out amyloidosis.

Our research findings and the results obtained by scientists from other countries indicate that superficial biopsies are often inadequate for diagnosing ATTR amyloidosis. To this end, in our patients with ATTR, amyloid deposits were not determined in the lamina propria of the mucous membrane. In GI amyloidosis, the lamina propria was affected in 78% of cases across different types of amyloidosis, while within the muscularis mucosae and submucosa, amyloid deposits were found in 89% of cases.

In their study of 146 GI biopsy samples, Hagen et al. found AL amyloid in the lamina propria in 30.7% of cases and in the muscularis mucosae in 41.6%. AA amyloid was frequently found in the lamina propria, while transthyretin amyloid was rare, detected in only 4.6% of lamina propria samples and 9.1% of muscularis mucosae samples [1]. These findings were consistent with the results of our research.

Similar data were presented in the study of 542 GI amyloidosis cases by Freudenthaler S. et al. It demonstrated that the lamina propria involvement was most prevalent in AA amyloidosis (90.1%), followed by AL kappa (89.2%), and AL lambda (79.4%) amyloidosis, while in ATTR amyloidosis, the frequency of the lamina propria involvement was the lowest (15.4%) [17].

In 14 autopsy cases of systemic amyloidosis, Marjanne den Braber-Ymker observed that amyloid deposits in the duodenum were more frequently found in the muscularis mucosae and submucosa in AL amyloidosis, while in AA amyloidosis, they were predominantly located in the lamina propria of the duodenal mucosa [26].

To improve the diagnostic accuracy of duodenal amyloidosis detection through biopsy, it is crucial to obtain samples from deeper tissue layers, in addition to the lamina propria, but endoscopists are often hesitant to perform such biopsies of deep duodenal tissues due to the increased risk of bleeding.

However, in a multifactorial analysis by Uraiwan W et al., no significant difference was found in the risk of bleeding complications in patients with and without amyloidosis undergoing biopsies (5.6% and 4.2%, respectively), even when considering various factors like age, gender, medical history, medications, biopsy site, lab results, and prior coagulopathy treatments. The presence of amyloidosis itself did not elevate the risk of bleeding during biopsies [27].

## 5. Conclusions

In our research, AL amyloidosis was found to be the most prevalent type of amyloidosis, affecting 54% of patients in the study cohort. In patients with AL kappa amyloidosis, the presence of the most intense interstitial and intravascular amyloid deposits was observed, potentially correlating with a faster and more severe clinical progression.

GI bleeding is a more common complication of AA amyloidosis due to the specific way amyloid proteins deposit in the GI tract, affecting, in addition to the deep tissue layers, the lamina propria of the mucous membrane. These amyloid deposits occurring in interstitial and intravascular spaces may lead to vascular fragility and increased friability.

When diagnosing AL lambda and ATTR amyloidosis with duodenal biopsies, superficial biopsies that only include the mucosal tissue can lead to false negative results, because amyloid deposits in these conditions tend to be concentrated in the deeper layers of the intestinal wall. This is particularly true for ATTR amyloidosis, where mucosal involvement is rare.

Moreover, in our series of autopsy studies, it was found that amyloidosis was diagnosed during life only in 37.5% of patients. This finding highlights the significant challenges associated with early diagnosis of this condition.

## Figures and Tables

**Table 1:** Types of amyloidosis affecting the gastrointestinal tract.

Type of Amyloidosis	Precursor Protein	Systemic	Localized
AL	Immunoglobulin light chain	✓	✓
AH	Immunoglobulin heavy chain	✓	✓
AA	Serum amyloid A	✓	-
ATTR	Transthyretin, wild type, Transthyretin, variants	✓	-
AApoAIV	Apolipoprotein A IV, wild type	✓	-
Aβ2M	β2-microglobulin, wild type β2-microglobulin, variants	✓	-
ALys	Lysozyme, variants	✓	-
ALECT2	Leukocyte chemotactic factor-2	✓	-
AFib	Fibrinogen α, variants	✓	-
AGel	Gelsolin, variants	✓	-
ASom	(Pro)somatostatin	-	✓
ACatK	Cathepsin K	-	✓
AEFEMP1	EGF-containing fibulin-like extracellular matrix protein 1 (EFEMP1)	-	✓

**Table 2:** Correlation of amyloidosis types with patient age and gender in the duodenal biopsy and autopsy samples.

Type of Amyloidosis	Patients, n		Age					Gender, n			
			Mean Age, Years			Age Range, Years		M		F	
	A	B	Total	A	B	A	B	A	B	A	B
AL-lambda	7	5	67.8	68.3	67	50–75	68–84	3	3	4	2
AL-kappa	1	7	63.3	68	63.1	68	53–93	0	4	1	3
AA	6	7	65.1	71.8	62.3	53–87	32–73	3	6	3	1
ATTR	2	2	65.7	91	58	90–91	48–68	0	2	2	0
Total	16	22		74.8	62.8	50–91	32–93	6	15	10	6
Total	37		65.4			32–93		21		16	
A-autopsy; B-biopsy											

## Data Availability

All data and materials are available upon reasonable request. Address requests to Z.G. (email: gioeva_z@mail.ru) or L.M. (email: mikhalevalm@yandex.ru), Avtsyn Research Institute of Human Morphology of Federal State Budgetary Scientific Institution “Petrovsky National Research Centre of Surgery”, Moscow, Russian Federation.

## Abbreviations

The following abbreviations are used in this manuscript:

ATTR	Transthyretin amyloidosis
GI	Gastrointestinal
H&E	Hematoxylin and eosin
IHC	Immunohistochemistry
